# Historic insights and future potential in wheat elaborated using a diverse cultivars collection and extended phenotyping

**DOI:** 10.1038/s41598-025-13678-w

**Published:** 2025-08-28

**Authors:** Khaoula El Hassouni, Muhammad Afzal, Philipp H. G. Boeven, Jost Dornte, Michael Koch, Nina Pfeiffer, Franz Pfleger, Matthias Rapp, Johannes Schacht, Monika Spiller, Malte Sielaff, Stefan Tenzer, Patrick Thorwarth, C. Friedrich H. Longin

**Affiliations:** 1 State Plant Breeding Institute, University of Hohenheim, Fruwirthstr. 21, 70599 Stuttgart, Germany; 2 Limagrain GmbH, Salder Str. 4, 31226 Peine-Rosenthal, Germany; 3 Deutsche Saatveredelung AG (DSV), Leutewitz 26, 01665 Kaebschuetztal, Germany; 4 KWS Lochow GmbH, Zuchstation Wetze, 37154 Northeim, Germany; 5 DIGeFa GmbH, Schützenberg 10, 32756 Detmold, Germany; 6 W. Von Borries-Eckendorf GmbH & Co. KG (WvB), Hovedisserstr. 94, 33818 Leopoldshöhe, Germany; 7 Institute for Immunology and Research Center for Immune Therapy (FZI), University Medical Center of the Johannes Gutenberg University Mainz, Langenbeckstr. 1, 55131 Mainz, Germany

**Keywords:** Genetics, Plant sciences

## Abstract

Wheat is one of the most important staple crops worldwide. Wheat breeding mainly focused on improving agronomy and techno-functionality for bread or pasta production, but nutrient content is becoming more important to fight malnutrition. We therefore investigated 282 bread wheat cultivars from seven decades of wheat breeding in Central Europe on 63 different traits related to agronomy, quality and nutrients in multiple field environments. Our results showed that wheat breeding has tremendously increased grain yield, resistance against diseases and lodging as well as baking quality across last decades. By contrast, mineral content slightly decreased without selection on it, probably due to its negative correlation with grain yield. The significant genetic variances determined for almost all traits show the potential for further improvement but significant negative correlations among grain yield and baking quality as well as grain yield and mineral content complicate their combined improvement. Thus, compromises in improvement of these traits are necessary to feed a growing global population.

## Introduction

Wheat (*Triticum aestivum* subsp. *aestivum*) ranks among the most important staple crops with a global growing area of about 219 million ha and a total grain production reaching 808 million tons in 2022^1^. This globally important food crop for human consumption provides a high nutritional value with starch, proteins, minerals, fibers and vitamins^2,3^. Given its prevalence in human diets, wheat cultivars must meet particular quality criteria for the production of different types of end-products. To meet the industrial requirements, wheat cultivars are classified in many countries based on baking quality as part of the registration process in the official seed catalogue. In Germany, this classification system includes four classes from highest to lowest quality corresponding to E, A, B and C class, respectively^4^. The classification into quality classes depends on minimum standards in seven quality traits.

Baking quality of wheat is related to the rheological properties of the dough, the dough strength and extensibility, which are largely determined by the protein content and its quality^5^. Around 80% of the wheat protein belongs to the gluten, which forms a unique dough network under input of water and kneading energy^6,7^. Therefore, wheat cultivars were often investigated with different tests regarding protein or gluten content and quality, dough characteristics as well as bread loaf volume^8^. Positive correlations between protein content and protein quality measured as sedimentation volume and loaf volume of bread were reported^9–11^.

A successful wheat cultivar must combine acceptable end-use quality with high grain yield and low susceptibility against diseases and lodging in the field with a reduced need for chemical plant protection. Unfortunately, protein content appears to have a strong negative correlation with grain yield^12,13^. Thus, breeders had to find a balanced way of selecting on these traits using innovative approaches, for instance, the grain-protein deviation, which became popular in Central Europe during the last decade^14^. Comparing results from official registration trials of wheat cultivars in Germany from 1984 to 2014, Laidig et al.^11^ and Voss-Fels et al.^15^ have shown large improvements mainly in grain yield and disease resistance and to a limited extent for end-use quality. With the high demand for healthy food nowadays and the worldwide health problems of micronutrient deficiencies known as hidden hunger^16^, it is also worthwhile to implement nutritional traits in the wheat supply chain^17,18^. For instance, the International Maize and Wheat Improvement Center (CIMMYT) in partnership with HarvestPlus have launched a large wheat breeding program which shall combine good agronomy and end-use quality with increased content of the minerals Fe and Zn in developing countries^17^. However, these efforts need to be extended to the global wheat supply chain. Until now, studies investigating the potential of combining nutrient content with better agronomical performance and end use quality are completely lacking for the important wheat production region of Central Europe.

Therefore, we investigated 282 bread wheat cultivars from seven decades of wheat breeding in Central Europe on 63 traits related to agronomy, baking and nutritional quality across up to eight field environments. Our objectives were to (i) quantify the variance due to cultivar, environment and cultivar-by-environment interaction for the investigated traits, (ii) explore the phenotypic correlations between the 63 traits, (iii) investigate the temporal trends in traits realized across seven decades of wheat breeding, and (iv) elaborate the potential to combine agronomical attributes and baking quality with nutritional quality to feed the global population.

## Results and discussion

In this study, we investigated 63 different traits across 282 wheat cultivars (Supplementary Data 1) originating from different European countries and different decades of wheat breeding tested under multiple field environments. Besides classical agronomic traits like grain yield, plant height and susceptibility of wheat cultivars against diseases, commonly measured quality traits like protein content, sedimentation volume and falling number, we dived deeply into techno-functional traits for dough and bread making quality as well as nutrient content measuring dozens of minerals and sugars. To our knowledge, this data set is one of the largest experimental data sets ever reported in wheat breeding representing a perfect basis to elaborate the historic and future potential of wheat breeding to better feed the growing world.

### Prerequisites of successful breeding fulfilled for the most agronomic, quality and nutritional traits

Very important prerequisites for successful breeding of a new trait are: (1) a significant difference in the trait between the existing wheat cultivars, i.e. significant genetic variance, (2) a high heritability, i.e. the amount of genetic variance compared to the overall phenotypic (visible) variance, (3) speed of measuring the trait across several samples, and (4) feasibility to combine a new trait with the other traits for selection, i.e. the new valuable trait expression should not be negatively correlated with the other traits.

**Fig. 1:**
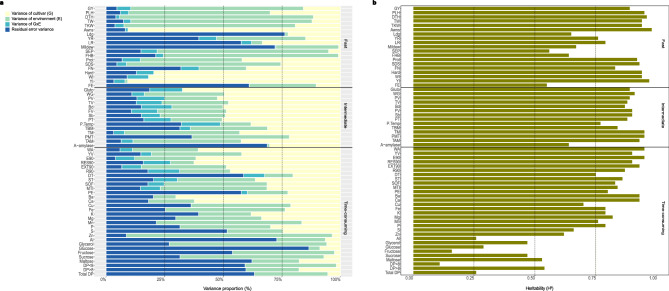
Variance components and heritability for all the measured traits on 282 bread wheat cultivars across mutiple environments. (**a**) Percentage of the phenotypic variation explained by each variance component for all traits. (**b**) Heritability for all measured traits. Horizontal lines indicate traits categories depending on the speed of the test. For a detailed description of the traits, see Methods section and Table 1.

For all investigated traits except lodging and oligosaccharides of the class DP < 8, we found a significant genetic variance (Fig. 1a, Supplementary Data 2), but its magnitude relative to the other sources of variation varied considerably across traits. It ranged from 0.8% of total variance for susceptibility against the fungal disease powdery mildew to 90.8% for yellow index measuring the yellow colour of the extracted flour. By contrast, the variance of cultivar-by-environment interaction was quite low for most traits indicating that the ranking of cultivars was quite stable across the different test environments. This confirms previous findings in the literature for those of the 63 traits measured in our study, which have been already reported in the literature^11,19,20^. Due to budgetary constraints, we had to reduce the number of samples for measuring the nutritional traits (see Methods section). Beyond others, we eliminated the replicated samples within environments and, thus, cultivar-by-environment variance could not be quantified for nutritional traits.

**Table 1:** Description of all the measured traits, measurement method and test speed.

Trait abbreviation	Trait name and measurement unit	Measurement method	Traits categories by test speed
GY	Grain yield (dt/ha)	Combine harvester at 14% moisture	Fast
PLH	Plant height (cm)	Height in cm from the ground to tip of the ears	
DTH	Days to heading (number of days)	Number of days when 60% of the plants per plot showing the first spikelets	
TW	Test weight (kg/hl)	Near-infrared spectroscopy (NIRS)	
TKW	Thousand kernel weight (g)	The weight of 1000 grains	
Awns	Awns (0/1)	Visual scoring in field (0) for absent and (1) for present	
Ldg	Lodging (1–9)	Visual scoring in field from 1–9 (absence – severe lodging)	
YR	Yellow rust (1–9)	Visual scoring of disease in field from 1–9 (healthy – highly susceptible)	
LR	Leaf rust (1–9)		
Mildew	Powdery Mildew (1–9)		
SEP	Septoria (1–9)		
FHB	Fusarium head blight (1–9)		
Prot	Protein content (%)	Dumas method—ICC 167	
SDS	Sedimentation test (ml)	Sediment in test tube—ICC 151	
FN	Falling number (s)	Perten falling number system—ICC 107/1	
Hard	Hardness	Near-infrared spectroscopy (NIRS)	
WI	White index	Konica Minolta chromameter CR-410 for flour color measurement	
YI	Yellow index		
FE	Flour extraction rate (%)	Flour with type 550/used kernels *100 with Brabender Quadrumat Junior mill	
Gluto	Glutograph	Brabender Glutograph	Intermediate
WG	Wet gluten	Perten Glutomatic	
PV	Peak1 (cP)	Rapid visco-analyzer-4 Newport scientific—ICC 162	
TV	Trough viscosity (cP)		
Bd	Breakdown (cP)		
FV	Final Viscosity (cP)		
Sb	Setback (cP)		
PT	Peak maximum time (min)		
P.Temp	Pasting Temperature (°C)		
TBM	Torque before maximum (BU)	Brabender Glutopeak 803,400	
TM	Torque maximum (BU)		
PMT	Peak maximum time (s)		
TAM	Torque after maximum (BU)		
A-amylase	Alpha-amylase activity SD unit/g	Enzymatic test kit—Megazyme α-Amylase SD Assay Kit (K-AMYLSD)	
WA	Water absorption (ml/100 g flour)	Farinograph—ICC115/1	Time-consuming
YV	yield of volume (ml/100 g flour)	Rapid-Mix Test—Rapeseed displacement according to Neumann/Doose	
E90	Energy (cm^2^) at 90 min	Brabender Extensograph E—ICC114/1	
RES90	Resistance to Extension (BE) at 90 min		
EXT90	Extensibility (mm) at 90 min		
R90	Ratio		
DT	Development time (min)	Perten micro-doughLAB 2800—ICC 184	
ST	Stability (min)		
SOF	Softening (mNm)		
MTI	Mixing Tolerance Index (mNm)		
PE	PeakEnergy (Wh/kg)		
Ba	Barium (mg/kg)	Inductively coupled plasma combined with optical emission spectroscopy (ICP‐OES) and mass spectrometry (ICP‐MS) for mineral content quantification	
Ca	Calcium (mg/kg)		
Cu	Copper (mg/kg)		
Fe	Iron (mg/kg)		
K	Potassium (mg/kg)		
Mg	Magnesium (mg/kg)		
Mn	Manganese (mg/kg)		
P	Phosphorus (mg/kg)		
S	Sulphur (mg/kg)		
Zn	Zinc (mg/kg)		
Al	Aluminum (mg/kg)		
Glycerol	Glycerol (mg/g)	High-performance anion-exchange chromatography with pulsed amperometric detection (HPAEC) for sugars quantification	
Glucose	Glucose (mg/g)		
Fructose	Fructose (mg/g)		
Sucrose	Sucrose (mg/g)		
Maltose	Maltose (mg/g)		
DP < 8	Oligosaccharide with degree of polymerization < 8 (mg/g)		
DP > 8	Oligosaccharide with degree of polymerization > 8 (mg/g)		
Total DP	Total oligosaccharide (mg/g)		

Regarding full decomposition of phenotypic variance, large magnitude of the variance due to environment stands out for almost all traits (Fig. 1a). For instance, average grain yield across all cultivars tested at single locations ranged from 73 dt/ha to 107 dt/ha over the six test environments although the agricultural practices and fertilizers’ supply were comparable (data not shown). This is most probably due to the micro-environmental differences between the different field environments, which is a well-known factor for its impact on numerous traits and multiple crops observed in breeders’ trials^11,19,21–23^. The significant cultivar-by-environment variance underlines the large importance of multi-location field trials in research and product development to estimate robust adjusted means and breeding values for traits of interest. Since many crops exist with hundreds of cultivars that can be grown across diverse environments, such experimental studies become expensive and large in terms of the number of test plots. However, they remain crucial for reliably generalizing results of the trials.

For majority of the investigated traits, heritability was higher than 0.6 (Fig. 1b). For some traits such as the element Al, most of the sugars and oligosaccharides, the heritability was below 0.5, which is to our knowledge for sugars yet not reported in literature for wheat but confirms findings from maize^24^. Compared to the estimates of heritability for frequently reported traits in literature such as grain yield^25^, plant height, disease susceptibility^26,27^ as well as quality traits like protein content and sedimentation volume^28,29^, our heritability estimates were high to very high. This can be explained by the large number of tested wheat cultivars expanding the genetic variance to the existing maximum and by the robust experimental set up using several field locations across two years allowing for meaningful conclusion for future wheat breeding. Summarizing, for many of the 63 traits, the prerequisite 1 and 2 of a significant genetic variance and a high heritability are fulfilled. For further discussion and illustrations, we concentrate on traits with heritability > 0.4.

For an efficient selection, breeders must evaluate hundreds of different candidate lines to find the best compromise regarding all traits of interest, which requires methods and techniques to rapidly measure the traits of interest on several samples. Similarly, trading wheat samples and products along the supply chain at all stakeholders on certain traits requires also rapid technologies to measure these traits as recently indicated in an opinion paper for future supply chains^18^. We tried to summarize the 63 traits into three groups regarding speed of the test methods i.e., fast, intermediate and time-consuming. Although agronomic evaluations require testing across at least one field season, breeders are well equipped for doing the final measurement for yield, agronomy and disease susceptibility of hundreds of candidate lines within a day. In the near future, that might get even faster due to the use of drones or other unmanned aerial vehicles^30–32^. Therefore, we put these agronomic traits into the group with fast determination method. Similarly, more than 100 samples per day can be processed using the following kernel and quality tests: protein content via NIRs, sedimentation volume, falling number, kernel hardness and size. Intermediate speed with a few dozen samples a day could be realized with glutograph glutopeak, rapid-viscoanalyser, wet gluten and flour colour measurements. Baking tests, detailed rheological tests as well as measuring minerals and sugars with the reference methods are slow. Thus, there is urgent need to develop fast methods for these traits, which accurately predict the respective traits, for healthy nutrition with high-quality products in future^18^. For many minerals, the X ray fluorescence detection method predicts their content very precisely with the capability to measure between 50–100 samples per day making its use in wheat breeding or trading highly interesting^33,34^.

### Correlation network defines limits for the combination of different traits

**Fig. 2:**
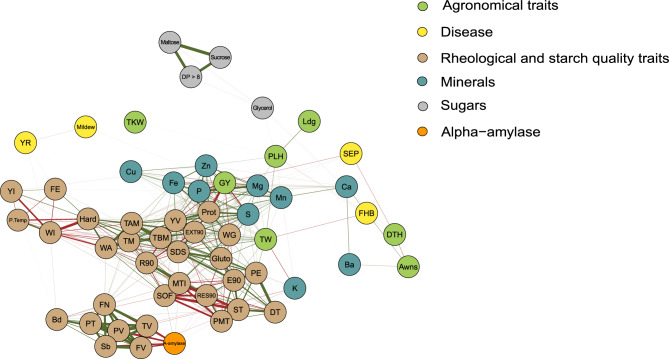
Correlation network of mean values across environments including significant associations among the traits for 282 wheat cultivars. Green and red lines connecting the nodes indicate positive and negative correlations, respectively. Thickness of the lines indicates the correlation strength. Correlations ranged from −0.86 to 0.98.

Coefficients of correlation among 58 traits were determined (Supplementary Data 3) and we tried to summarize their relationship in a network analyses (Fig. 2). At first glance, three distinct clusters of trait associations become visible: (1) three sugars and oligosaccharides, (2) falling number, α-amylase activity and the traits of the rapid-visco-analyser, and (3) a high number of traits comprising agronomic and quality traits as well as few minerals. Cluster 2 represents all direct or indirect measurements of starch quality performed in our study. Rapid-visco analyser measures pasting properties of a flour–water suspension, which are largely influenced by the starch properties of the examined flour^35^. With increased amount and activity of α-amylase, starch is degraded to smaller sugar components impacting pasting properties, which is indirectly measured by falling number test^36,37^. Consequently, the clustering of these traits in our network analyses is logical and expected. Interestingly, starch properties are neither correlated with agronomic traits nor with dough quality traits to a higher extent. Thus, they could be combined without negative drawbacks with good agronomy or good dough and baking quality measured as loaf volume. This is of particular interest, as they determine important breadcrumb characters, which are not measured in baking tests routinely used in wheat variety testing in Germany.

Cluster 3 contains many traits but few subgroups are visible. For instance, loaf volume is highly positively correlated with protein content (r = 0.74, Supplementary Data 3) but also to protein quality as determined by SDS (r = 0.51, Supplementary Data 3). Furthermore, several parameters of dough quality tests were found to be positively correlated with protein content and protein quality, e.g. glutograph, extensograph (E90, EXT90), glutopeak (TBM, TM, TAM) and micro-doughLAB (PE). This is in line with findings in the literature for these traits^11,28^.

Protein content is also positively correlated with most measured minerals with the largest coefficient of correlation of r = 0.73 to S content (Supplementary Data 3), which confirms previous studies^38,39^. S content is correlated (r > 0.45) with loaf volume, SDS, EXT90 and glutograph. This reasonably strong association of S with dough and baking quality might be either due to the correlation with protein content, or by the fact that disulfide bonds are crucial for the establishment of the gluten network in a dough^40,41^ or both of it.

The single trait kernel hardness was correlated (r > 0.5) with water absorption, EXT90, SDS, TM, TBM, TAM (all glutopeak parameters), flour extraction rate and yellow index of the extracted flour. Whereas, the coefficient of correlation of the kernel hardness with white index of the extracted flour and pasting temperature from RVA was < 0.5 (Supplementary Data 3). Kernel hardness in wheat is largely influenced by the puroinduline genes^42^ Pin-a (Pina-D1b) and Pin-b (Pinb-D1b), which can be determined by kompetitive allele specific PCR (KASP) technology^43^. While Pina-D1b is fixed in EU wheat germplasm to the soft wild type allele as confirmed in our study (data not shown), we could observe variation in the tested wheat cultivars for Pinb-D1b segregating for soft wild type allele (a) and three different hard alleles (b, c, d; Supplementary Figure 1). The large impact of the soft allele at Pinb-D1b was especially pronounced for kernel hardness, SDS, water absorption, pasting temperature and colour of the extracted flour (Supplementary Figure 1), while the different hardness alleles had only a minor effect on these traits. These findings fit well with a previous study on 94 German wheat cultivars^29^ and highlight the high potential of molecular (marker-based) selection if loci with large effect on individual traits are known. Therefore, a more detailed investigation of the genetic architecture of the investigated traits in that study is of interest and will be elaborated in a future publication.

Owing to the demand to feed a growing world limited in the agricultural area, quality and nutrient content must be combined with high grain yield. Unfortunately, grain yield and protein content are strongly negatively correlated as often shown in literature^11,12,44^ and confirmed once again in our data set (r = - 0.78). Even more, grain yield and loaf volume as well as grain yield and the concentration of several minerals are also negatively correlated (Fig. 2, Supplementary Data 3). Consequently, improving these traits in parallel is complicated requiring priority settings and strategies to find best compromises for the future world nutrition. While numerous approaches were reported for combined selection of high grain yield and high protein content, e.g. protein yield or grain protein-deviation; cf. Thorwarth et al.^14^, strategies for the combination of high grain yield with high baking quality and high nutritional value are rare. In current wheat breeding in Germany, nutritional traits are not regarded and individual breeders work differently applying linear multi-trait indices, selection of independent culling levels (i.e. all candidate lines with disease susceptibility > 5 are discarded regardless their yield and quality potential) up to “visual selection” across many traits in big excel sheets. Thereby, choice of index type and the weighting of trait can largely influence gain of selection^45^ and depends on factors such as market situation of the breeder, expected future political or stakeholder regulations and other requirements expected in five—ten years. As farmers received only little price primes on quality the last decade in Germany, breeding focused on highest grain yield at a certain intermediate quality level (low A quality class). By contrast, due to political restrictions for nitrogen fertilization, we speculate that future interest will address also high quality with acceptable high yield.

In conclusion, the high genetic variance coupled with a high heritability for most of the investigated traits would enable efficient wheat breeding for better agronomy, baking quality as well as mineral content of future wheat cultivars. However, the slow speed of test methods especially for baking quality and some minerals as well as negative correlations between grain yield, baking quality and mineral content makes their efficient selection and combination difficult.

### Large historic breeding success on agronomy and partly on baking quality

**Fig. 3:**
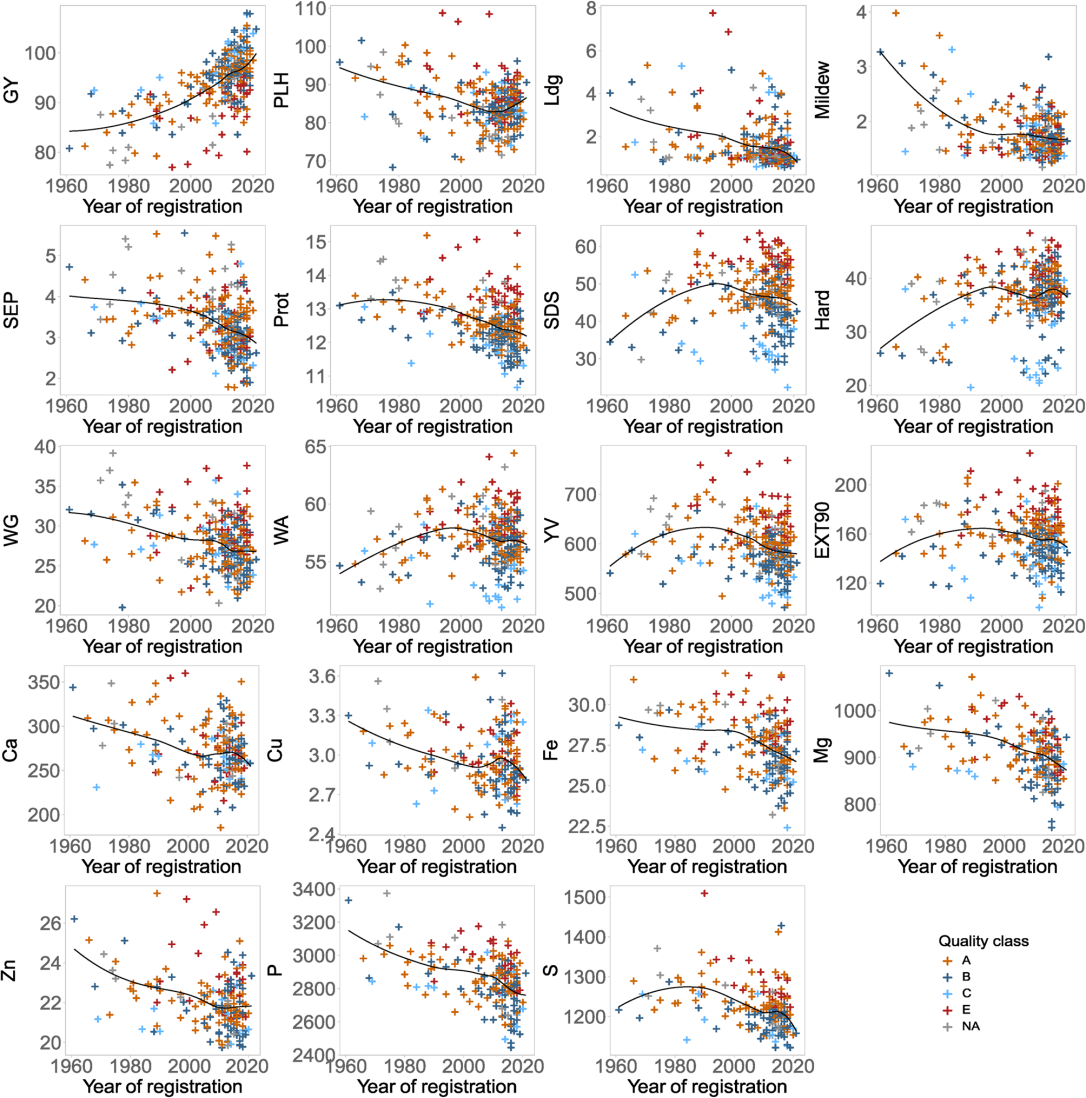
Scatterplot of different traits showing a temporal trend according to the registration year of the wheat cultivars. Locally estimated scatterplot smoothing (LOESS) regression is represented in black.

The tested 282 wheat cultivars were registered between 1961 and 2020. The historic wheat cultivars were chosen based on their large market relevance during the respective decades and seed availability, but most of the investigated wheat cultivars were registered after 2000 (Supplementary Data 1). Owing to that unbalanced data structure, we focus on important and large trends and used the LOESS regression accounting for that imbalance. For grain yield, an average increase from 81 dt/ha to almost 100 dt/ha was observed for wheat cultivars from 1961 and 2020, respectively (Fig. 3). Moreover, important agronomic traits like reduced susceptibility against fungal diseases, e.g. Septoria tritici blotch or powdery mildew, as well as reduced risk of lodging due to reduced plant height were achieved by breeding, which are of utmost importance if future agriculture will be based on less agrochemical inputs. These findings are in line with previous studies for German^11,15^ and international wheat germplasm^46^ underlining significant success and importance of plant breeding for feeding the growing world population under climate change adopting more sustainable agricultural practices.

Protein and wet gluten content were lower in modern than in historic wheat cultivars, but the reduction from 1961 to 2020 was much less pronounced than expected by their large and negative correlation with grain yield and the tremendous increase of grain yield in the same period. This highlights the breeders’ strategy to improve grain yield and in parallel efforts to avoid large reductions of protein content. Additionally, although negatively correlated to grain yield, trends of selection for traits related to protein quality, e.g. SDS, EXT90, or loaf volume increased over time until ~ 1990 and appeared to stagnate or slightly decreased afterwards. These results confirm previous studies^47,48^ and underline that even negatively correlated traits can be jointly improved.

**Fig. 4:**
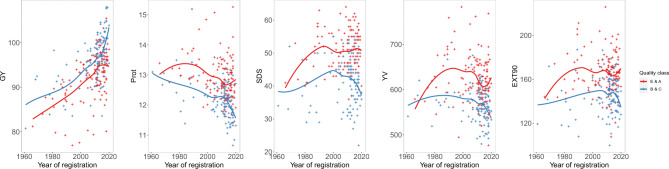
Scatterplot of different traits with a dual temporal trend according to the registration year of the wheat cultivars. Locally estimated scatterplot smoothing (LOESS) regression is represented in red for cultivars with E and A quality class and in blue for cultivars with B and C quality class.

Interestingly, the different wheat cultivars clustered for grain yield, protein content and baking quality across the decades also according to their quality classes (E > A > B > C baking quality class referring to the German reference system) as visible by the different coloured dots in Fig. 3. Therefore, we divided the wheat cultivars into two groups (Fig. 4): high baking quality (E + A quality cultivars) and limited baking quality (B + C quality cultivars) making several important findings observable. First, breeding for high quality was based on higher protein content at the expense of reduced grain yield. Second, while quality remained constant on average or even declined in wheat cultivars belonging to B + C quality group, it was increased up to 1990 and then kept constant in the E + A quality group. Third, a large variation in grain yield and quality traits became visible in all quality groups. This variation enables not only breeders but also the stakeholders along the supply chain to choose the cultivar with desired compromise, e.g. the highest yield within a respective quality class or the highest quality at a certain yield level.

Since the pioneering work of Payne et al.^49^, the monitoring and selection of certain high-molecular weight protein subunits became feasible and was intensively used by breeders. In particular, the alleles at glutenin locus Glu-1 at the homologous chromosomes 1 A, 1B and 1D were intensively monitored and selected. Therefore, we determined them across all 282 wheat cultivars by classical gel-electrophoresis (Supplementary Data 4 and Supplementary Figure 2). Following trends across registration years appeared: at Glu-A1 locus, breeders reduced the frequency of allele “0” by increasing the frequencies of alleles “1” and “2*”. Similarly, at Glu-B1, the frequency of allele “6 + 8” was reduced and the frequency of allele “7 + 9” was increased. Interestingly, the frequencies of these alleles varied considerably across the quality classes. Compared to cultivars from low baking quality class C, cultivars from highest baking quality class E had higher (lower) frequencies of alleles “1” and “2*” (“0”) at Glu-A1, higher (lower) frequencies of alleles “7 + 8” and “7 + 9” (“6 + 8”) at Glu-B1 and higher (lower) frequencies of alleles “5 + 10” (“2 + 12”) at Glu-D1 (Supplementary Figure 2). This is a further proof of the effectiveness of selection on single genes or gene families, as long as they have a large effect on a trait like also discussed above for the Puroinduline gene and kernel hardness.

Regarding mineral content, the trend across registration years was decreasing for almost all of them. Although European wheat breeders have not yet selected for or against minerals, this trend was expected due to the negative correlation between grain yield and mineral content (Fig. 2, Supplementary Data 3). Nevertheless, researchers from CIMMYT in partnership with the HarvestPlus program have shown that good agronomy and baking quality could also be combined with increased mineral content. Similarly, we see a clear tendency in our data set that wheat cultivars of better-quality groups, e.g. E class, appear to have higher mineral content than cultivars of poor baking quality class C (Fig. 3, Supplementary Figure 3).

### Outlook: Future breeding combines high mineral content, high baking quality and good agronomic preformance

**Fig. 5:**
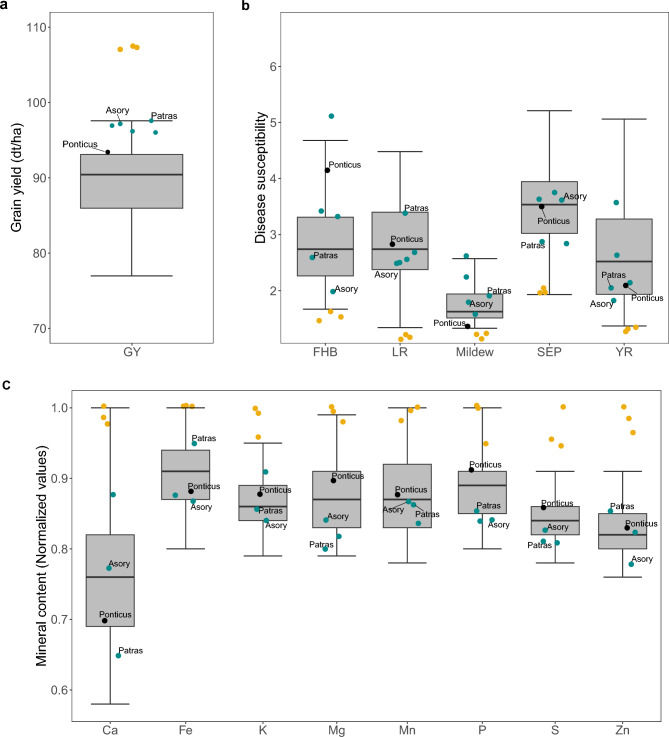
Boxplots showing the grain yield, disease response and content of nine minerals for the 20% best cultivars for loaf volume. The dots colored in blue are the top 5 cultivars within the 20% selection combining very good baking quality, high yield **(a)** with low disease susceptibility **(b)** and good nutritional value in minerals **(c)**. The dots colored in yellow are the best 3 cultivars in the whole panel for each individual trait. “Patras”, “Asory” and “Ponticus” are widely cultivated cultivars in Germany. In **(c)** , the values of mineral content were normalized between 0 and 1.

We therefore chose the wheat cultivars belonging to the 20% with the highest loaf volume and showed their variability in grain yield, disease susceptibility and mineral content in Fig. 5. The five cultivars with the highest yield in that group were marked with light blue across all boxplots and important market cultivars were shown with their names. Overall, a large variability for all regarded traits was present in that group of cultivars with high loaf volume showing the potential to combine high grain yield, good disease susceptibility, good baking quality and elevated mineral content. For instance, the cultivar “Patras” with significant market share for several years in Germany had a grain yield of 97.6 dt/ha, disease susceptibility from 1.5 for Mildew to 3.5 for LR and concentration of Fe and Zn in the upper half of tested wheat cultivars. As visible by the best wheat cultivars for individual traits (yellow dots in Fig. 5), however, the combination of different negatively correlated traits requires a compromise at the expense of reduced individual trait values. Thus, the weight given to the individual traits in the final combination must be elaborated carefully^45^ and might differ across world regions and time warranting further research.

Nevertheless, the large expenses and complications with negative correlations to grain yield in wheat breeding for higher mineral content are only justifiable, if consumers eat in future much more whole grain products with adopted bread making processes. This is due to the fact that the majority of minerals is in the outer layers and embryo of wheat grains, which are removed during milling of the widely used extracted flour. Additionally, minerals like Fe and Zn are bound in wheat flour to phytic acid, making them non bio-available for humans. During the bread making process, however, phytic acid can be largely degraded making minerals bioavailable by longer dough fermentation and (natural) addition of the enzyme phytase^50^.

## Conclusion

With our recent global challenges to feed a growing world population sustainably under a changing climate, malnutrition, and the large impact of our diet on chronic diseases^51^, it appears worthwhile and urgent to make impactful investments across supply chains for improving yield, sustainable agricultural production and nutrient content for as many crops as possible^18^. Therefore, our study investigating 63 traits related to agronomic performance, baking quality and nutrients in 282 wheat cultivars grown across up to eight environments is an important first step for future European wheat breeding. We could show substantial genetic variance and high heritability for most of the traits, which are essential for effective breeding. However, negative correlations between grain yield, quality and nutritional traits present challenges for parallel improvement. The historical breeding success suggests that strategic selection can partly mitigate these challenges. From a practical breeding perspective, understanding trait relationships is crucial since improving one trait can negatively affect others. This highlights the importance of selecting for multiple traits in a balanced way. The integration of genomic and phenomic predictions^52^ hold great potential to help breaking down this complexity and accelerating breeding progress. Recently, spectroscopy-based predictions of dough and baking properties showed encouraging results^53,54^ and recent developments in high-throughput proteomics and metabolomics coupled with AI might even improve that. The faster the prediction method and the lower amount of seed/plant material is needed for it, the easier it is to implement it into early generation selection, where relatively low prediction abilities would already allow to separate the better from the poor candidate lines. Thus, further research is warranted to (1) establish rapid methods to measure nutrients and complicated dough and baking qualities, (2) deepen our understanding of the genomics behind the different traits in order to establish marker-based selections and/or being able to break up negative correlations of traits, and (3) attract more interest by the stakeholders and consumers towards healthy diets and their realization across global supply chains.

## Methods

### Plant material and field experiments

This study evaluated a diverse panel of 282 old and modern winter wheat cultivars originating from different European countries. The cultivar choice was led by the wheat breeders involved in that project aiming to represent the diversity regarding genomics and quality of wheat breeding in Germany from 2000 −2021. Furthermore, few recent important wheat cultivars of neighbouring countries as well as few very important wheat cultivars from the last six decades of wheat cropping were added, as long as sufficient seeds were available. The tested cultivars were released between 1961 and 2021. Most of the cultivars were released in Germany (for more details see Supplementary Data 1). The entire panel underwent field-testing in two consecutive cropping seasons in yield and observation trials across Germany (Supplementary Data 5). Yield trials were carried out at locations: Hovedissen (51°59′12.8"N, 8°44′54.4"E), Leutewitz (51°8′35.3"N, 13°25′9.0"E) and Seligenstadt (49°51′3.30’'N, 10°05′21.60’'O) in 2020, and at Hovedissen, Asendorf (52°6′4.722"N, 9°1′35.79"E) and Seligenstadt in 2021. Observation trials were conducted in years 2020 and 2021 at locations Hohenheim (48°43′07.3"N, 9°11′08.7"E), Hovedissen, Leutewitz and Wetze (51°44′24.0"N, 9°54′36.0"E). In total, there were six environments (three locations and two years) for yield trials and eight environments (four locations and two years) for observation trials. Both trials were conducted using a partially replicated (P-rep) design with 300 plots at each location with randomization performed using CycDesign software^55^. The experimental design included 282 cultivars, of which 18 were chosen arbitrarily by the software at each environment to be replicated and the remaining ones were unreplicated, resulting in a total of 300 plots per enviornment. The experimental layout followed a grid structure consisting of 12 blocks, each containing 25 plots. For yield trials plots size ranged from 7.2 to 13.4 m^2^ depending on the location and standard field practices according to the local agricultural practice were implemented for fertilizer, fungicide, herbicide and growth regulator applications. Observation trials were conducted in small plots of size between 0.5 to 1 m^2^ without any applications of fungicides or growth regulators.

### Phenotyping and reference analytics

The traits assessed in this study are described in detail in Table 1. Grain yield (GY) reported as dt/ha was recorded in yield trials by harvesting all plots with a combine harvester and adjusted to 14% moisture content. Days to heading (DTH) was recorded as days elapsing between the first day of the year until 60% of plants had emerged heads. Plant height (PLH) was measured in cm from the ground to tip of the spikes, excluding awns. Awns were recorded as either absent (0) or present (1). Lodging (Ldg) was measured shortly before harvest on a 1–9 scale, where 1 denotes absence of lodging and 9 severe lodging with plot completely flattened.

From the harvest of each plot, thousand kernel weight (TKW) and test weight (TW) were determined according to the variety registration regulations following DIN EN ISO 520 for TKW and DIN EN ISO 7971–3 for TW. In the observation trials, disease susceptibility of the plants was recorded for yellow rust (YR), leaf rust (LR), fusarium head blight (FHB), powdery mildew (Mildew) and Septoria tritici leaf blotch (SEP) at adult plant stage. All diseases were visually rated using a scale ranging from 1 to 9, where 1 refers to healthy and 9 to completely infected plants.

For detailed quality analyses, the harvested samples of the yield trials were used. Due to the extensive time and budget required for analyzing the quality traits, all samples from only four of six yield environments were used. These four environments (Seligenstadt in 2020, Hovedissen in 2020, Seligenstadt in 2021 and Asendorf in 2021; Supplementary Data 5) were chosen based on quality of the field trials and protein content, to avoid samples which would be deemed unacceptable in wheat trading due to too low protein contents, which is common practice for quality assessment in registration trials in Germany. The following quality parameters were analyzed according to the reference methods or standard methods of the International Association for Cereal Science and Technology (ICC): Grain protein content (Prot; ICC 167), SDS sedimentation volume (SDS; ICC 151), falling number (FN; ICC 107/1), Farinograph water absorption (WA; ICC 115/1), Extensograph (E90, EXT90, RES90, R90; ICC 114/1), Rapid Visco Analyser (PV, TV, Bd, FV, Sb, PT, P.Temp; ICC 162) and Micro-dough LAB (DT, ST, SOF, MTI, PE; ICC 184). Kernel hardness was measured by near infrared (NIR) spectroscopy. White and yellow indices (WI & YI) were determined as L and b* values reading on chromameter Konica Minolta using extracted flour. Dough and gluten properties were measured using the standard routines of the Perten Glutomatic for wet gluten, Brabender Glutograph (a kind of fats method for Extensograph) and the Brabender GlutoPeak (measuring dough built up in an aequeous-flour suspension kneaded constantly for a given time) according to the manufacturer’s instructions. The baking test was performed according to the Rapid Mix Test (RMT)^56^. This is the gold standard baking test for investigating baking quality in cultivar trials in Germany. Briefly, 1 kg of extracted flour and the water amount according to Farinograph measurement for the respective flour are used with 50 g yeast, 15 g salt, 10 g sugar, 10 g fat and 20 ppm ascorbic acid, very intensively mixed and 30 bread rolls are formed. After proofing and baking the loaf volume is determined.

A subset of 800 samples, consisting of 200 samples from the four environments (Seligenstadt in 2020, Hovedissen in 2020, Seligenstadt in 2021 and Asendorf in 2021), were analyzed for mineral elements and sugars. These 200 cultivars are a subset of the original panel of 282, selected to include major cultivars while capturing genetic diversity. The reduction from 282 to 200 cultivars was due to budget constraints. Therefore, for mineral elements and sugars analyses, samples were not replicated within environments, and as a result, cultivar by environment interaction could not be estimated for these traits. Mineral content of whole grain flours was determined in mg/kg by inductively coupled plasma combined with optical emission spectroscopy (ICP‐OES) and mass spectrometry (ICP‐MS) without technical replication. Values below the detection limit of 0.025 mg/kg were excluded. Using whole grain flour of each sample, sugars, sugar alcohols and oligosaccharides were quantified by High-performance anion-exchange chromatography with pulsed amperometric detection (HPAEC) technique (sugar alcohols, mono- and disaccharides adapted from Thermo Fisher Scientific Technical Note 72,225; oligosaccharides according to Thermo Fisher Scientific Application Note 1149) after aqueous extraction without technical replication in the lab^57^. Alpha-amylase analysis was performed using the α-Amylase SD Assay Kit^58^ (Megazyme, Wicklow, Ireland).

Electrophoretic analysis was performed to identify high molecular weight glutenin subunits (HMW-GS) composition of the wheat cultivars used in this study. Protein extraction was carried out according to Osborne^59^ followed by disulphide bond reduction with Dithiothreitol. HMW-GS were separated by sodium-dodecyl-sulphate polyacrylamide gel electrophoresis (SDS-PAGE). The HMW-GS were identified using the previously proposed nomenclature by Payne and Lawrence^60^.

The kernel hardness in wheat is predominantly controlled by Puroindoline genes Pin-a (Pina-D1b) and Pin-b (Pinb-D1b). Allele specific PCR markers were used to screen different mutations in the Puroindoline genes PinaD1 and PinbD1 following the protocol described by Mohler et al.^29^. The KASP maker for Pin-a and Pin-b was developed in house by commercial breeding partner. It was run using the KASP-TF MasterMix (LGC Group, Middlesex, UK) following manufacturers protocol.

### Data analysis

All statistical computations were performed with the R software^61^ (R Development Core Team 2018) and software package ASReml-R 3.0^62^. In the statistical analyses, each environment represents the location-by-year combination. Phenotypic data analysis was performed according to the following statistical model, given in Eq. (1):

1 $${y}_{ikno}=u+{g}_{i}+{env}_{k}+{g}_{i}:{env}_{k}+{rep}_{kn}+{b}_{kno}+{e}_{ikno}$$

where y_ikno_ was the phenotypic observation for the *ith* cultivar tested in the *kth* environment in the *nth* replication in the *oth* incomplete block, u was the general mean, g_i_ the genotypic effect of the *ith* cultivar, env_k_ the effect of the *kth* environment, g_i_ : env_k_ was the cultivar-by-environment interaction, rep_kn_ was the effect of the *nth* replication in the *kth* environment, b_kno_ was the effect of the *oth* incomplete block of the *nth* replication in the *kth* environment and e_ikno_ was the residual.

Outliers were detected by initially assessing plot quality (through observation and comments). Diagnostic plots were then used for preliminary detection followed by standardization of residuals using alternative outliers mixed models (aom) in ASReml^63^.

Variance components were estimated using the restricted maximum likelihood (REML) method assuming a random model in a classical one-stage analysis. The significance of random terms was tested by model comparison using a likelihood ratio test^64^. Average values across environments were estimated as Best Linear Unbiased Estimates (BLUEs) assuming fixed genotypic effects. Broad sense heritability (H^2^) was calculated according to the following Eq. (2)^65,66^:

2 $${H}^{2}=1-\frac{\vartheta }{{2\sigma }_{G}^{2}}$$

where ϑ is the mean variance of a difference of two best linear unbiased predictors and ${\sigma }_{G}^{2}$ the genetic variance.

Pearson correlation coefficients were estimated for all traits using the BLUEs across environments and the R package ‘corrplot’^67^. Correlation network analysis was performed using the R package ‘qgraph’^68^. To test whether the measured traits have a temporal trend across breeding periods (registration years of cultivars), we used the LOESS regression by fitting a smooth curve through a scatterplot to account for the unbalanced number of cultivars per decade. Box-and-whisker plots were constructed by ggplot2 package^69^ using the BLUEs across environments. The values in Fig. 5c were normalized as follows:

$$x=\frac{{x}_{i}}{Max\_x}$$

where x_i_ is the trait observation value of a cultivar and Max_x is the trait maximum value. 

## Supplementary Information

Supplementary Information 1.

Supplementary Information 2.

Supplementary Information 3.

Supplementary Information 4.

Supplementary Information 5.

Supplementary Information 6.

## Data Availability

The datasets used and analyzed during the current study available from the corresponding author on reasonable request. Analyzed data is provided within the manuscript or supplementary information files.
