# Supplementary material for: Historic insights and future potential in wheat elaborated using a diverse cultivars collection and extended phenotyping
Source: Sci Rep. 2025 Aug 28;15:31674. doi: 10.1038/s41598-025-13678-w (PMC12391296; doi:10.1038/s41598-025-13678-w)
Supplement: Supplementary file 6 — Supplementary Information 6. [file 41598_2025_13678_MOESM6_ESM.docx]

**Supplementary material**

**Historic insights and future potential in wheat elaborated using a diverse cultivars collection and extended phenotyping.**

Khaoula El Hassouni^1^*****, Muhammad Afzal^1^, Philipp H. G. Boeven^2^, Jost Dörnte^3^, Michael Koch^3^, Nina Pfeiffer^4^, Franz Pfleger^5^, Matthias Rapp^6^, Johannes Schacht^2^, Monika Spiller^4^, Malte Sielaff^7^, Stefan Tenzer^7^, Patrick Thorwarth^1^, C. Friedrich H. Longin^1^*****

^1^State Plant Breeding Institute, University of Hohenheim, Fruwirthstr. 21, 70599 Stuttgart, Germany.

^2^ Limagrain GmbH, Salder Str. 4, 31226 Peine-Rosenthal, Germany

^3^Deutsche Saatveredelung AG (DSV), Leutewitz 26, 01665 Kaebschuetztal, Germany

^4^KWS Lochow GmbH, Zuchstation Wetze 37154 Northeim, Germany

^5^DIGeFa GmbH, Schützenberg 10, 32756 Detmold, Germany

^6^W. von Borries-Eckendorf GmbH & Co. KG (WvB), Hovedisserstr. 94, 33818 Leopoldshöhe, Germany

^7^Institute for Immunology and Research Center for Immune Therapy (FZI), University Medical Center of the Johannes Gutenberg University Mainz, Langenbeckstr. 1, 55131 Mainz, Germany

*** Correspondence:**

Khaoula El Hassouni

Email: khaoula.elhassouni@uni-hohenheim.de

C. Friedrich H. Longin

Email: friedrich.longin@uni-hohenheim.de

**Supplementary figures**

**
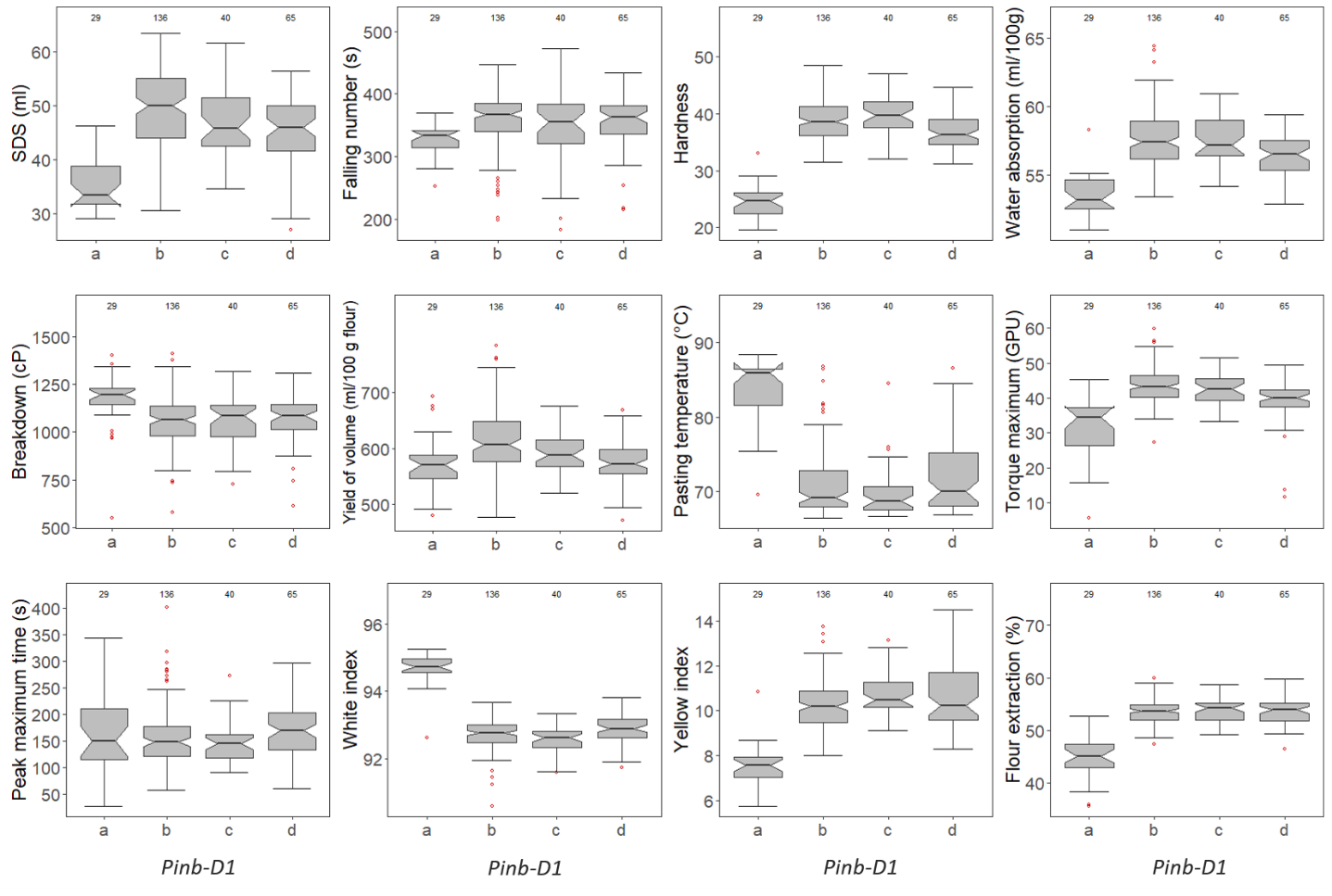
Supplemntary Figure 1.** Boxplots showing the effect of different alleles (a, b, c, d) of the *Puroindoline b-D1 gene (Pinb-D1)* on different traits for 282 bread wheat cultivars. The allele a is the soft type and the alleles b,c,d are three different alleles inheriting hard type. ** Supplemntary Figure 2.** Frequency of high molecular weight glutenins in bread wheat genotypes according to different quality classes E, A, B, and C.

**Supplemntary Figure 3.** Scatterplot of different traits showing a temporal trend according to the registration year of the wheat genotypes. LOESS regression represented in black.

**Legends for Supplementary Tables**

**Supplementary Data 1 (separate file).** List of the 282 wheat cultivars used in this study, their country of origin, year of registration and quality class.

**Supplementary Data 2 (separate file).** Summary statistics of all measured traits among 282 bread wheat cultivars. Var G genotypic variance, Var E variance of environment, Var GxE genotype-by-environment interaction variance, H² heritability.

**Supplementary Data 3 (separate file).** Pearson correlation matrix of all measured traits.

**Supplementary Data 4 (separate file).** Allelic diversity of glutenin composition and frequency of high molecular weight glutenins in bread.

**Supplementary Data 5 (separate file).** Locations used for field testing and their basic characteristics.
